# Training time frame, a possible key to literature discrepancy on fasting athletes

**DOI:** 10.1186/s12970-016-0154-7

**Published:** 2016-11-24

**Authors:** Navid Moghadam, Farzin Farahbakhsh

**Affiliations:** 1 Sports Medicine Department, Rasool Akram Hospital, Iran University of Medical Sciences, Tehran, Iran; 2 Sports Medicine Research Center, Tehran University of Medical Sciences, Tehran, Iran

**Keywords:** Athletes, Fasting, Athletic performance

## Abstract

There is a discrepancy in the literature as to whether fasting during Ramadan affects athletic performance.

We suggest in this letter that the time frame for training is a confounding variable that has been missed in the literature.

## Main text

Fasting during the month of Ramadan is a common practice among Muslim athletes who are adherent to Islamic code. According to Islamic code, healthy adult Muslims (except for women during menstruation) should abstain from any consumption from dawn to sunset in Ramadan month; they can only break the fast after sunset. There are several studies that have examined the effect of fasting during Ramadan on exercise performance.

Zarrouk et al. studied the neuromuscular performance and anthropometric parameters of athletes in the time of Ramadan fasting; they concluded that Ramadan fasting did not decrease neuromuscular performance nor did it affect anthropometric parameters of fasted athletes [1]. Their findings were in contrast with studies that showed the negative effect of Ramadan Fasting in athletes.[2] Zerrouk et al. suggested that these discrepancies might be related to mood and motivations of athletes or their sleep and calorie intake.

Kordi et al. studied two groups of athletes with different training Time frame. They compared athletes who train while they are still fasting with athletes who hold their training sessions after breaking fast at sunset. In this study they concluded that Ramadan fasting could have a positive effect on performance in athletes who do train after breaking fast in comparison to the other group [3].

These findings suggest that the training time frame (before or after breaking fast at sunset during Ramadan) could be considered as a possible confounding variable in assessing the effect of Ramadan fasting on athletic performance which previous studies did not pay enough attention to [1, 2].

Improvement in athletes’ performance who trained after breaking fast at sunset during Ramadan fasting [3] is a novel finding which can lead to improvement in nutrition strategies in Muslim athletes and even in non-Muslims.

We suggest that future studies examine the time frame of training during Ramadan to get a better understanding of how it may potentially affect athletic performance.
